# Supplementary material for: Winner-takes-all resource competition redirects cascading cell fate transitions
Source: Nat Commun. 2021 Feb 8;12:853. doi: 10.1038/s41467-021-21125-3 (PMC7870843; doi:10.1038/s41467-021-21125-3)
Supplement: Supplementary file 1 — Supplementary Information [file 41467_2021_21125_MOESM1_ESM.pdf]

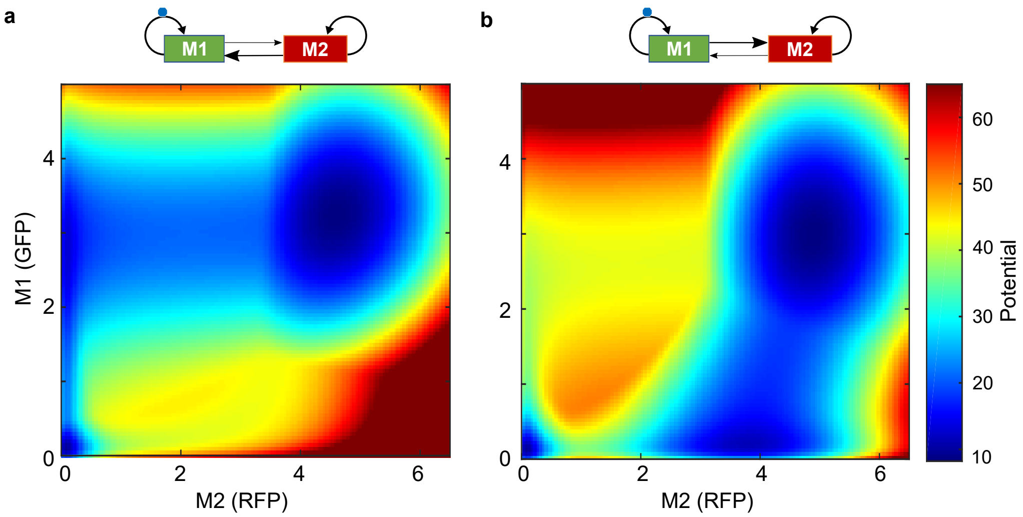

Supplementary Fig.1. Calculated potential landscape of the Syn-CBS circuit using the mathematical model without resource competition. The potential represents the stability of the steady states or the probabilities of the cells attracted to them (blue represents a higher probability while red represents a lower probability). **a** The scenario with a weak M1-to-M2 link and strong M2-to-M1 link. **b** The scenario with a strong M1-to-M2 link and weak M2-to-M1 link. This is an extension of Fig. 1.

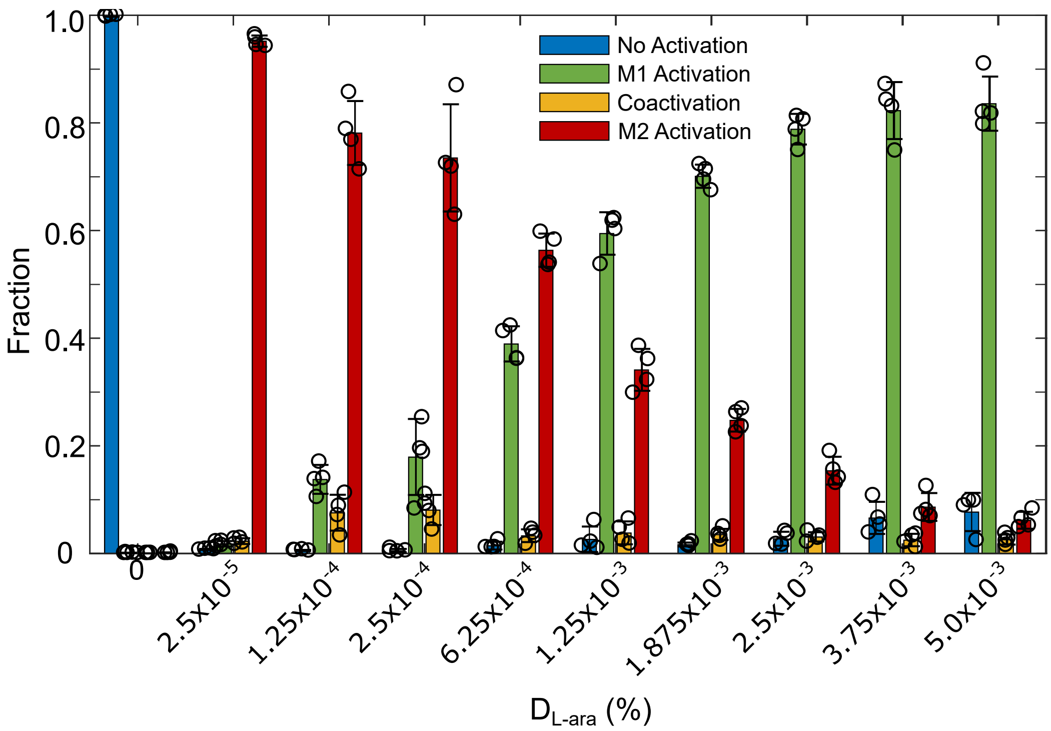

Supplementary Fig.2. The fraction of cells in different fates controlled by the one-strain Syn-CBS circuit with increasing dose of L-ara ($D_{L\text{-}ara}$). The fractions were estimated from flow cytometry data. Data displayed as mean ± SD (n = 4 biological independent samples). Circuit CT61 was used here. This is an extension of Fig. 2. Source data are provided as a Source Data file.

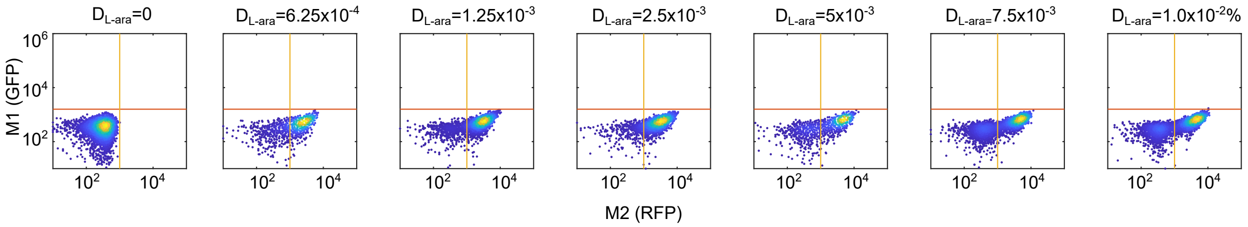

Supplementary Fig.3. One-strain Syn-CBS circuit with low-copy backbone is not able to activate Module 1 (M1) due to resource competition. Flow cytometry data shows cell state transitions in one-strain Syn-CBS circuit with low-copy backbone by increasing level of inducer L-ara ($D_{L\text{-}ara}$). 10,000 events were recorded for each sample. Data from one representative of four biological replicates. Circuit CT61 with low-copy backbone (pMMB206) was used here. Source data are provided as a Source Data file.

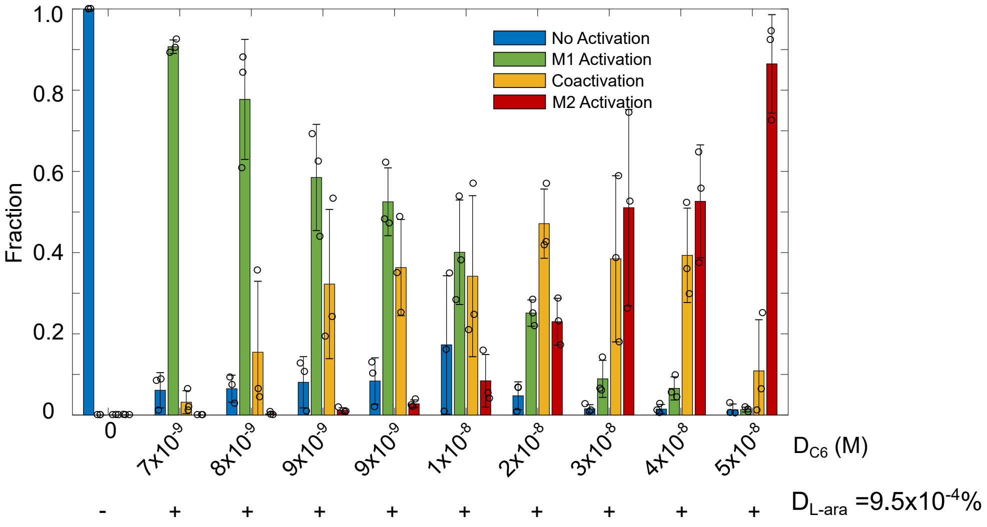

Supplementary Fig.4. The fraction of cells in different fates controlled by the two separated bistable switches system with increasing dose of C6 (D_{C6}) and a fixed dose of L-ara (D_{Lara}=9.5x10^{-4}%). The fractions were estimated from flow cytometry data. Data displayed as mean ± SD (n = 3 biological independent samples). Circuit IC15 was used here. This is an extension of Fig. 3. Source data are provided as a Source Data file.

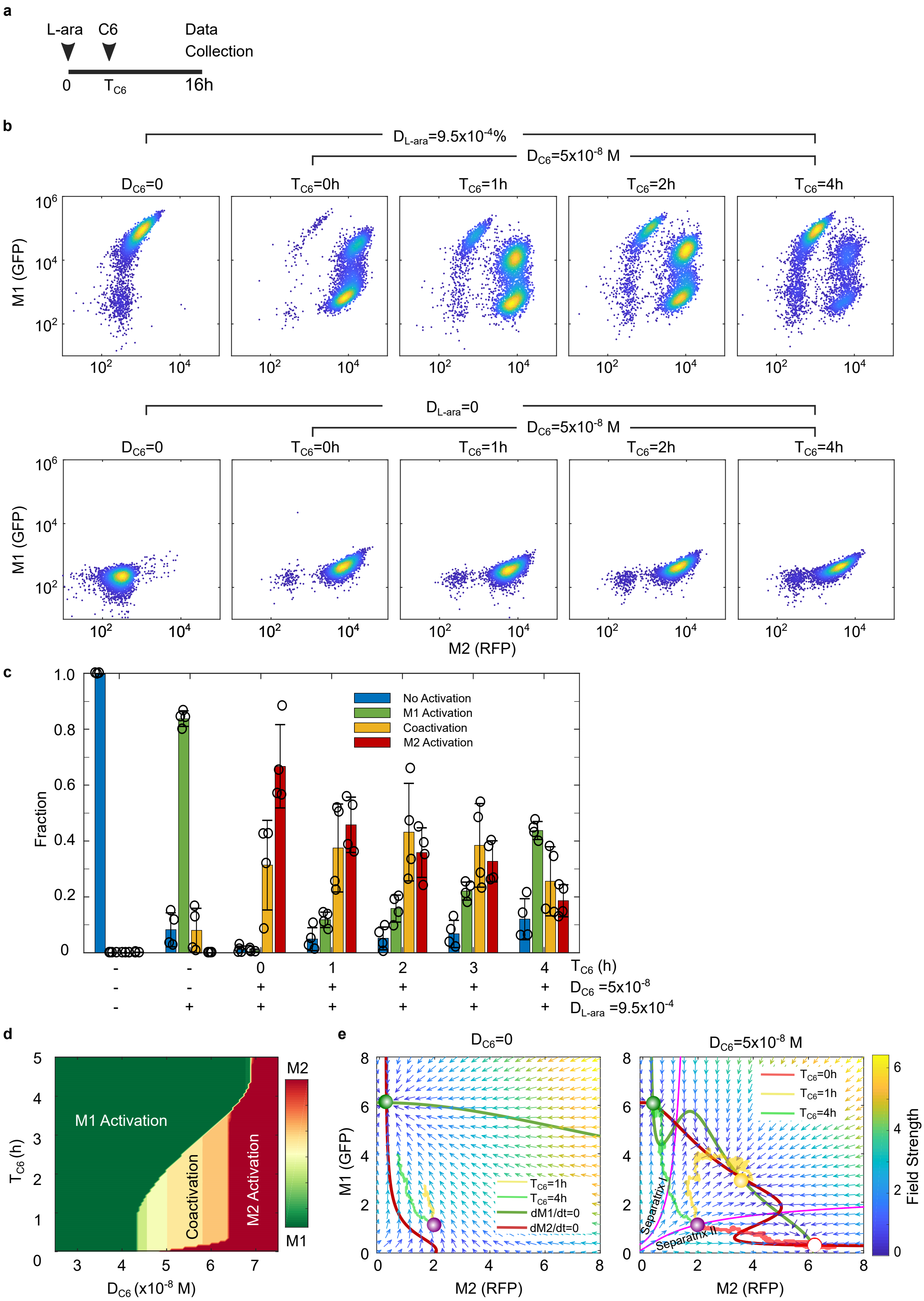

a
L-ara
C6
Data Collection
0
T_C6
16h
b
D_L-ara=9.5x10^-4%
D_C6=5x10^-8 M
D_C6=0
T_C6=0h
T_C6=1h
T_C6=2h
T_C6=4h
M1 (GFP)
D_L-ara=0
M2 (RFP)
c
Fraction
No Activation
M1 Activation
Coactivation
M2 Activation
T_C6 (h)
D_C6 =5x10^-8
D_L-ara =9.5x10^-4
d
M1 Activation
Coactivation
M2 Activation
M2
M1
T_C6 (h)
D_C6 (x10^-8 M)
e
D_C6=0
D_C6=5x10^-8 M
M1 (GFP)
M2 (RFP)
T_C6=1h
T_C6=4h
dM1/dt=0
dM2/dt=0
T_C6=0h
Separatrix I
Separatrix II
Field Strength

Supplementary Fig.5. Resource competition between two separate bistable switches with sequential addition of the two inducers. a Diagram of the experimental design. The doses of L-ara and C6 were fixed. L-ara was added at the time 0, and C6 was added at various time points. D_{C6} and T_{C6} mean the dose and the time of the addition, respectively, of C6. **b** Flow cytometry data for experiments described in Fig. 5a showed cell state transitions. 10,000 events were recorded for each sample. Data from one representative of four biological replicates. **c** The fractions of cells in different fates controlled by the two separated bistable switches system with various T_{C6}. The fractions were estimated from flow cytometry data. Data displayed as mean ± SD (n = 4 biological independent samples). **d** Simulated cell fates in the space of the dose and timing of inducer C6. L-ara dose was fixed as $D_{L\text{-}ara}$=9.5x10^{-4} %. **e** Simulated stochastic trajectories in the phase plane diagram. The initial state of the cells is set to the steady-state without any inducer (purple circle). The nullclines of M1 and M2 are shown in green and red, respectively, while separatrices are shown in pink. The three cell fates (red, green, and yellow circles) are found at the intersections of the two nullclines. The vector field of the system is represented by small arrows, where the color is proportional to the field strength. Three representative single-cell stochastic trajectories (highlighted green, yellow, and red) show the evolution of the system from the same initial condition ($D_{L\text{-}ara}$=0% and D_{C6}=0 M) to three different states with various T_{C6}. The dose of C6 was fixed as D_{C6}=0 M in the left panel and D_{C6}=5x10^{-8} M in the right panel. L-ara was fixed as D_{Lara}=9.5x10^{-4} % in both panels. Circuit IC15 was used here. Source data are provided as a Source Data file.

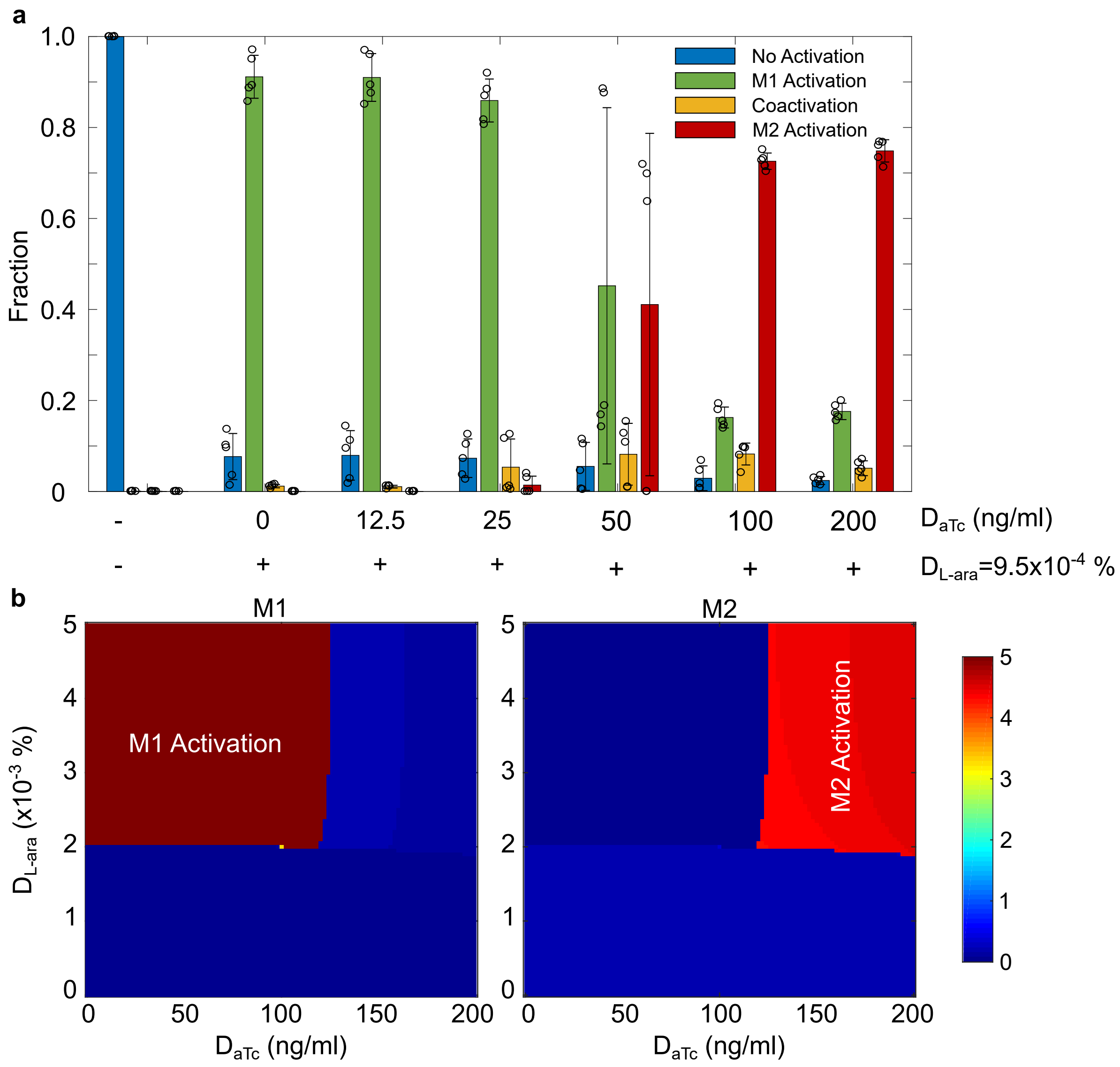

Supplementary Fig.6. Tune the outcomes of the resource competition by controlling relative strength of module connections. a The fractions of cells in different fates controlled by the Syn-CBS circuit (circuit CT81) by increasing the dose of aTc (D_{aTc}). The fractions were estimated from flow cytometry data. Data displayed as mean ± SD (n = 5 biological independent samples). **b** Simulated cell fates in the doses space of two inducers L-ara and aTc. Source data are provided as a Source Data file.

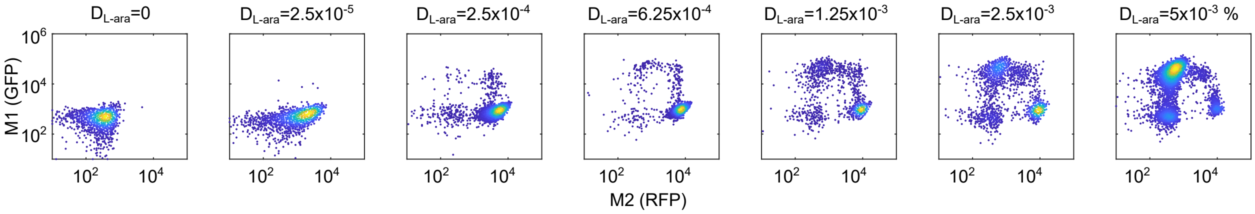

Supplementary Fig.7. One-strain Syn-CBS circuit with hybrid promoter but without TetR module confirmed the resource competition between two modules. Flow cytometry data shows cell state transitions with various doses of inducer L-ara ($D_{L\text{-}ara}$). 10,000 events were recorded for each sample. Data from one representative of four biological replicates. Circuit IC25 was used here. Source data are provided as a Source Data file.

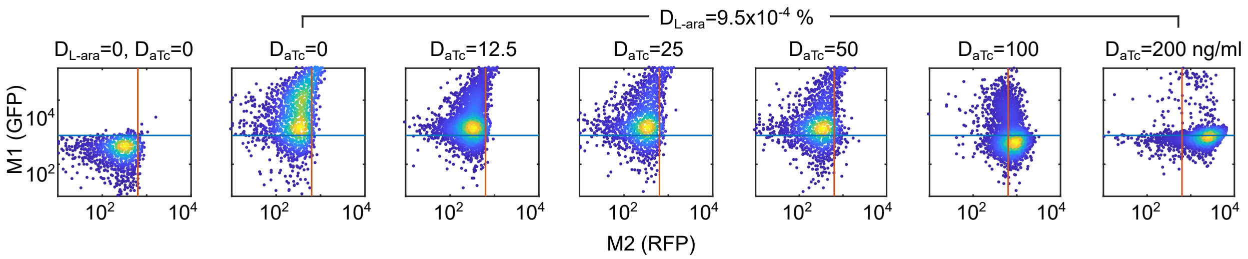

Supplementary Fig.8. One-strain Syn-CBS circuit with low-copy backbone and TetR module confirmed the resource competition between two modules. Flow cytometry data shows cell state transitions with various doses of inducer aTc (D_{aTc}) and a fixed dose of L-ara ($D_{L\text{-}ara}$). 10,000 events were recorded for each sample. Data from one representative of three biological replicates. Circuit CT81 with low-copy backbone (pMMB206) was used here. Source data are provided as a Source Data file.

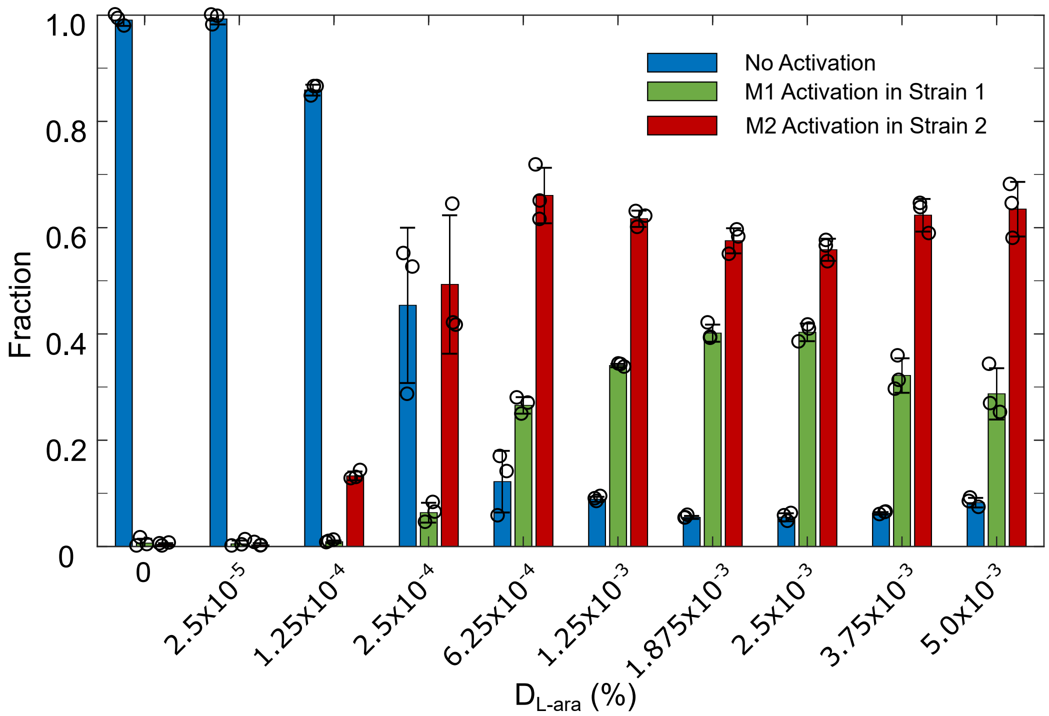

Supplementary Fig.9. The fraction of cells in different fates as a function of inducer L-ara controlled by the two-strain Syn-CBS circuits without the TetR module. The fractions were estimated from flow cytometry data. Data displayed as mean ± SD (n = 3 biological independent samples). This is an extension of Fig. 5. Circuits CT66 and CT67 were used here. Source data are provided as a Source Data file.

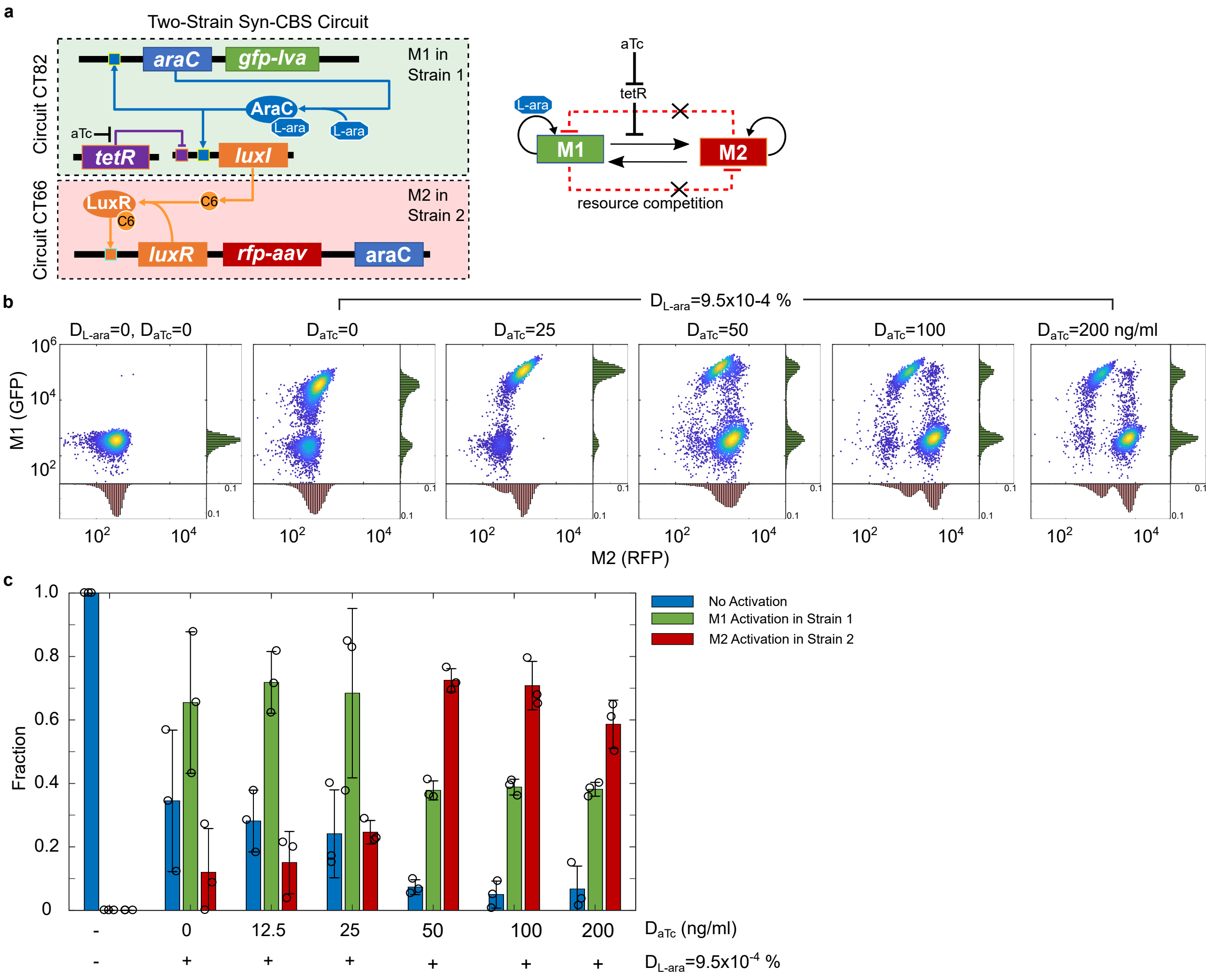

Supplementary Fig.10. Mitigation of resource competition with microbial consortia. a Diagram of two-strain Syn-CBS circuits with a TetR module. **b** Flow cytometry data showed the expected stepwise cell state transitions by increasing the dose of aTc (D_{aTc}, ng/mL) in the two-strain Syn-CBS circuit with the TetR module. 10,000 events were recorded for each sample. Data from one representative of three biological replicates. **c** The fraction of cells in different fates as functions of inducer aTc. The fractions were estimated from flow cytometry data. Data displayed as mean ± SD (n = 3 biological independent samples). Circuits CT66 and CT82 were used here. Source data are provided as a Source Data file.

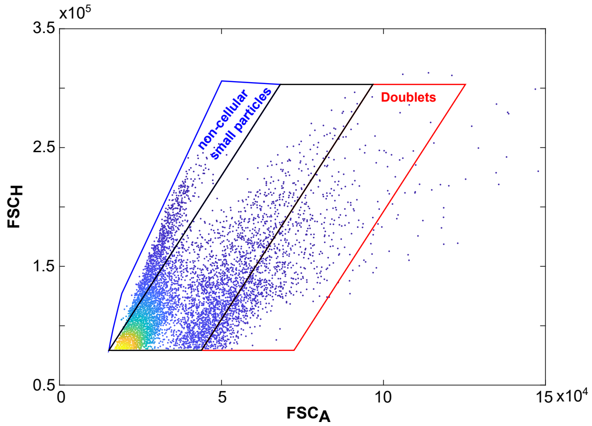

Supplementary Fig.11. Gating strategy for all the Flow Cytometry Data. Cells were gated using FSC-A/FSC-H to eliminate the doublets (red region) and non-cellular small particles (blue region) according to data from the plain LB medium without any cells as a negative control. The data points in the black box are used in all the flow cytometry data analysis.

Supplementary Table 1. BioBrick parts used in this paper.

BioBrick number	Abbreviation in the paper	Description
K206000	Pbad	Inducible promoter activated by AraC and L-arabinose
C0061	LuxI	3-oxo-C6-HSL producing enzyme
C0040	TetR	Tetracycline repressor from transposon Tn10
J23116	Pcon	Constitutive promoter
J04031	GFP	GFP with LVA tag
B0034	RBS	Ribosome binding site
B0015	Terminator	Transcriptional terminator (double direction)
pSB1C3	pSB1C3	High copy (100-500 copies) BioBrick assembly backbone with chloramphenicol resistance
pSB3K3	pSB3K3	Medium copy (20-30 copies) BioBrick assembly backbone with kanamycin resistance

Supplementary Table 2. List of monocistronical operons.

ID	Description (promoter-gene)	sub-parts (promoter+RBS+gene+terminator)	Backbone
op9	Pbad-araC	K206000+B0034+araC+B0015	pSB1C3
K750000	Pbad-gfpLVA	K206000+B0034+K145915+ B0015	pSB1C3
op12	Pbad-luxRG2C	K206000+B0034+luxRG2C+ B0015	pSB1C3
op97	Plux9-rfpAAV	Plux9+B0034+rfpAAV+ B0015	pSB1C3
op105	Plux9-luxRG2C	Plux9+B0034+luxRG2C+ B0015	pSB1C3
op101	Plux9-araC	Plux9+B0034+araC+ B0015	pSB1C3
op127	J23116-tetR	J23116+B0034+P0440+ B0015	pSB1C3
op111	Pbad/tet-luxI	Pbad/tet+B0034+C0061+ B0015	pSB1C3
op54	Pbad-luxI	K206000+B0034+C0061+ B0015	pSB1C3

Supplementary Table 3. List of gene circuits.

ID	Assembly from operons	Promoter-gene Description	Backbone
CT61	K750000+op9+op105+op97+op101+op54	Pbad-GFPlva+Pbad-araC+Plux9-luxRG2C+Plux9-RFPaav+Plux9-araC+Pbad-luxI	pSB3K3
CT81	op127+op111+K750000+op9+op105+op97+op101	J23116-tetR+PBad/tet-luxI+Pbad-GFPlva+Pbad-araC+Plux9-luxRG2C+Plux9-RFPaav+Plux9-araC	pSB3K3
CT66	op105+op97+op101	Plux9-luxRG2C+Plux9-RFPaav+Plux9-araC	pSB3K3
CT67	K750000+op9+op54	PBADs-GFPlva+PBADs-araC+PBADs-LuxI	pSB3K3
CT82	op127+K750000+op9+op111	J23116-tetR+Pbad-GFPlva+Pbad-araC+Pbad/tet-luxI	pSB3K3
IC15	K750000+op9+op105+op97	Pbad-GFPlva+Pbad-araC+Plux9-luxRG2C+Plux9-RFPaav	pSB3K3
IC25	op111+K750000+op9+op105+op97+op101	PBad/tet-luxI+Pbad-GFPlva+Pbad-araC+Plux9-luxRG2C+Plux9-RFPaav+Plux9-araC	pSB3K3

Supplementary Table 4. Stochastic models for the synthetic gene circuits.

Reaction	Description	Propensity function
$\Phi \rightarrow M1$	Basal production rate of M1	$(v_{01} \cdot R_{01})/PF_Q \cdot \Omega$
$\Phi \rightarrow M1$	Production rate of M1	$(v_1 \cdot R_1)/PF_Q \cdot \Omega$
$M1 \rightarrow \Phi$	Degradation rate of M1	$d_1 \cdot M1$
$\Phi \rightarrow M2$	Basal production rate of M2	$(v_{02} \cdot R_{02})/PF_Q \cdot \Omega$
$\Phi \rightarrow M2$	Production rate of M2	$(v_2 \cdot R_2)/PF_Q \cdot \Omega$
$M2 \rightarrow \Phi$	Degradation rate of M2	$d_2 \cdot M2$

For the Syn-CBS circuit with resource competition:

$$R_1 = \frac{Sa \cdot AraC^2}{Sa \cdot AraC^2 + \Omega^2} \cdot N_{cp}, \quad R_2 = \frac{Su \cdot LuxR^2}{Su \cdot LuxR^2 + \Omega^2} \cdot N_{cp}, \quad Sa = C_{min1} + (C_{max1} - C_{min1}) \cdot \frac{L0^n}{L0^n + J1^n},$$

$$Su = C_{min2} + (C_{max2} - C_{min2}) \cdot \frac{C6^m}{C6^m + J2^m}, \quad R_{01} = N_{cp}, \quad R_{02} = N_{cp}, \quad AraC = M_1 + M_2 \cdot \lambda_2,$$

$$C6 = M_1 \cdot \lambda_1, LuxR = M_2, PF_Q = (\frac{1}{Q_{01}} + \frac{1}{Q_{02}} + \frac{R_1}{Q_1} + \frac{R_2}{Q_2}) + 1.$$

For the two separate switches (Syn-SBS) system with resource competition:

$$R_1 = \frac{Sa \cdot AraC^2}{Sa \cdot AraC^2 + \Omega^2} \cdot N_{cp}, \quad R_2 = \frac{Su \cdot LuxR^2}{Su \cdot LuxR^2 + \Omega^2} \cdot N_{cp}, \quad Sa = C_{min1} + (C_{max1} - C_{min1}) \cdot \frac{L0^n}{L0^n + J1^n},$$

$$Su = C_{min2} + (C_{max2} - C_{min2}) \cdot \frac{C6^m}{C6^m + J2^m}, \quad R_{01} = N_{cp}, \quad R_{02} = N_{cp}, \quad AraC = M_1,$$

$$LuxR = M_2, \quad PF_Q = \left(\frac{1}{Q_{01}} + \frac{1}{Q_{02}} + \frac{R_1}{Q_1} + \frac{R_2}{Q_2}\right) + 1.$$

Supplementary Information

Mathematical model for the Syn-CSB circuit without considering resource competition

The Syn-CBS circuits are composed of two modules. In each module, there is one activator, which promotes its own production, thus forming a self-activation motif. Specifically, in Module 1 (M1), the AraC-L-ara dimer binds to promoter P_{bad} to promote the production of itself, reporter GFP, and the signal C6 for Module 2. In Module 2 (M2), the LuxR-C6 dimer binds to the promoter P_{lux} to induce the production of itself, reporter RFP, and another copy of araC. Thus, the two modules promote each other. The construction of the model for the AraC self-activation module (M1) is based on our previous works[1]. The LuxR self-activation module (M2) is similar to the AraC self-activation module and follows a similar equation. The connections of the two modules are mediated by the AraC-mediated production of C6 and the LuxR-mediated production of AraC. Here, for simplicity, we model the genes under the same promoter as one variable instead of modeling all the genes as separate variables. It is noted that the GFP and RFP are also the direct reporters of these two variables. That is, GFP shows M1 expression levels and RFP shows M2 expression levels. This simplification is reasonable given that the production rates for the genes under the same promoter should be similar as each operon constituting the circuits was constructed monocistronically. In this way, we can build a two-dimensional ordinary differential equations (ODEs) model with two variables, M_1 for the genes in Modules 1 and M_2 for the genes in Module 2. The level of C6 is based on M1 with one coefficient λ_1. The total level of AraC includes the part in M1 and the part mediated by LuxR that is based on M2 with one coefficient λ_2. This two-dimensional ODE model allows us to do nullcline and direction field analysis directly. The mathematical model for the Syn-CBS circuit can be simplified by the following two equations:

$$\frac{dM_1}{dt} = f_1(M_1, M_2) - d_1 \cdot M_1$$

$$\frac{dM_2}{dt} = f_2(M_1, M_2) - d_2 \cdot M_2$$

Where $\mathrm{f_1 = \left(k_{01} + k_1 \cdot \frac{Sa \cdot AraC^2}{Sa \cdot AraC^2 + 1}\right)}$, $\mathrm{Sa = C_{min1} + (C_{max1} - C_{min1}) \cdot \frac{Lara^n}{Lara^n + J_1^n}}$, $\mathrm{AraC = M_1 + M_2 \cdot \lambda_2}$, $\mathrm{f_2 = \left(k_{02} + k_2 \cdot \frac{Su \cdot LuxR^2}{Su \cdot LuxR^2 + 1}\right)}$, $\mathrm{Su = C_{min2} + (C_{max2} - C_{min2}) \cdot \frac{C6^m}{C6^m + J_2^m}}$, , $\mathrm{C6 = M_1 \cdot \lambda_1}$, and $\mathrm{LuxR = M_2}$. Here, k_{01} and k_{02} are the basal expression levels of M1 and M2, while k_1 and k_2 are the maximum production rates of M1 and M2, respectively. S_a describes how the production rate is regulated by inducer L-ara. Here, C_{max1} and C_{min1} are the maximum and minimum affinities of the

AraC dimers to the binding sites on the promoter P_{bad}. It is noted that f1 is a function of AraC that includes both M1 and M2, thus the positive autoregulation in Module 1 and the connection from Module 2 to Module 1 are formed. S_u describes how the production rate is regulated by the LuxR. Here, C_{max2} and C_{min2} are the maximum and minimum affinities of the LuxR dimers to the binding sites on the promoter P_{lux}. Similarly, f2 is a function of LuxR that include M2 and thus the positive autoregulation in Module 2 is formed. f2 is also a function of C6 that includes M1 and thus the connection from Module 1 to Module 2 is formed. As well, n represents the nonlinearity of the promoter activation by L-ara, and d_1 and d_2 are the degradation rates of the modules. The input of the system is the concentration of L-ara. The two reporters are $\mathrm{GFP} = \mathrm{M}_1$ and $\mathrm{RFP} = \mathrm{M}_2$. The model is suited to analyze the steady-state behavior of the system under conditions without resource competition. The theoretical analysis of the Syn-CBS circuit in Fig. 1 is based on this model. The fitted parameters are, unless otherwise mentioned: $\mathrm{C_{min1}} = 0.25$, $\mathrm{C_{max1}} = 2$, $\mathrm{J_1} = 6 * 10^{-3}$, $n = 3$, $\mathrm{k_{01}} = 0.1$, $\mathrm{k_1} = 4$, $\mathrm{d_1} = 1$, $\mathrm{C_{min2}} = 0.2$, $\mathrm{C_{max2}} = 2$, $\mathrm{J_2} = 1.5$, $m = 3$, $\mathrm{k_{02}} = 0.1$, $\mathrm{k_2} = 5$, $\mathrm{d_2} = 1$, and $\mathrm{Lara} = 1.2 * 10^{-3}$. The strength of the connections between the two modules are set as $\lambda_1 = 0.25$ and $\lambda_2 = 0.2$ in Fig.1b-c and $\lambda_1 = 0.5$ and $\lambda_2 = 0.03$ in Fig.1d-e to demonstrate two possible theoretical designs of the synthetic cascading bistable switches that correspond to differing module connection strengths.

Mathematical model for the Syn-CSB circuit when considering resource competition

The expectations demonstrated from the mathematical model of the Syn-CSB circuit without resource competition was not consistent with the experimental data, thus we developed a general mathematical model for a synthetic gene circuit by considering the resources (RNA polymerase and ribosome) in the host cell (see the following section below) and applying it to the Syn-CBS circuit. The mathematical model for the Syn-CSB circuit is thus revised as follows:

$$\frac{dM_1}{dt} = (v_{01} \cdot R_{01} + v_1 \cdot R_1)/PF_Q - d_1 \cdot M_1$$

$$\frac{dM_2}{dt} = (v_{02} \cdot R_{02} + v_2 \cdot R_2)/PF_Q - d_2 \cdot M_2$$

where $\mathrm{R_1} = \frac{\mathrm{Sa \cdot AraC^2}}{\mathrm{Sa \cdot AraC^2+1}} \cdot N_{cp}$, $\mathrm{R_2} = \frac{\mathrm{Su \cdot LuxR^2}}{\mathrm{Su \cdot LuxR^2+1}} \cdot N_{cp}$, Sa, Su, AraC, LuxR are defined as before and $\mathrm{PF_Q} = (\frac{1}{\mathrm{Q_{01}}} + \frac{1}{\mathrm{Q_{02}}} + \frac{\mathrm{R_1}}{\mathrm{Q_1}} + \frac{\mathrm{R_2}}{\mathrm{Q_2}}) + 1$. Compared to the above model without resource competition, this model has an additional denominator in the production rate, PF_Q, that is a function of R1 and R2 (see the following section on the general mathematical model for the synthetic circuit with resource competition). In the functions of R1 and R2, the levels of transcription factors AraC and LuxR depends on M1 and M2, respectively, thus creating mutual inhibition between the two

modules as a result of resource competition. Here, we now consider N_{cp} as the copy number of the plasmid. The theoretical analysis of the Syn-CBS circuit in Fig. 2 and Fig. 4 is based on this model. The copy number of the plasmid is in a range of 20-30 for our system. Thus, we used $N_{cp} = 24$ in the mathematical model. The fitted parameters are, unless otherwise mentioned, $\mathrm{C_{min1}} = 0.003$, $\mathrm{C_{max1}} = 0.1275$, $\mathrm{J_1} = 0.75 * 10^{-3}$, $n = 3$, $\mathrm{v_{01}} = 0.0005$, $\mathrm{v_1} = 0.5$, $\mathrm{d_1} = 0.25$, $\mathrm{C_{min2}} = 0.005$, $\mathrm{C_{max2}} = 0.175$, $\mathrm{J_2} = 0.5$, $m = 3$, $\mathrm{v_{02}} = 0.0025$, $\mathrm{v_2} = 0.5$, $\mathrm{d_2} = 0.25$, $\lambda_1 = 1.25$, $\lambda_2 = 0.044$, $\mathrm{Q_{01}} = 300$, $\mathrm{Q_1} = 300$, $\mathrm{Q_{02}} = 3$, $\mathrm{Q_2} = 3$, and $\mathrm{Lara} = 0 \sim 5 * 10^{-3}$. L-ara is set to $1.25 * 10^{-3}$ in Fig. 2e-f. The inducer aTc in the Syn-CBS circuit with the tetR module is set by the level of λ_1. aTc range is set to 0~200 by linearly scale $\lambda_1 = 0 \sim 1.25$ in Supplementary Fig. 6.

Mathematical model for the two separate switches system

The two separate switches system was used to verify resource competition between the two modules within the Syn-CBS circuit and the WTA behavior. Most parts of the system are the same as the Syn-CBS circuit except for the two links that connect the modules, including AraC-mediated production of C6 and LuxR-mediated production of AraC, that were removed in the two separate switches system. Thus, the mathematical model for the two separate switches system with resource competition is as follows:

$$\frac{dM_1}{dt} = (v_{01} \cdot R_{01} + v_1 \cdot R_1)/PF_Q - d_1 \cdot M_1$$

$$\frac{dM_2}{dt} = (v_{02} \cdot R_{02} + v_2 \cdot R_2)/PF_Q - d_2 \cdot M_2$$

where $\mathrm{R_1} = \frac{\mathrm{Sa \cdot AraC^2}}{\mathrm{Sa \cdot AraC^2 + 1}} \cdot N_{cp}$, $\mathrm{R_2} = \frac{\mathrm{Su \cdot LuxR^2}}{\mathrm{Su \cdot LuxR^2 + 1}} \cdot N_{cp}$, $\mathrm{Sa} = \mathrm{C_{min1}} + (\mathrm{C_{max1}} - \mathrm{C_{min1}}) \cdot \frac{\mathrm{L0^n}}{\mathrm{L0^n + J1^n}}$, $\mathrm{Su} = \mathrm{C_{min2}} + (\mathrm{C_{max2}} - \mathrm{C_{min2}}) \cdot \frac{\mathrm{C6^m}}{\mathrm{C6^m + J2^m}}$, $R_{01} = N_{cp}$, $R_{02} = N_{cp}$, $\mathrm{AraC} = \mathrm{M_1}$, $\mathrm{LuxR} = \mathrm{M_2}$, and $\mathrm{PF_Q} = (\frac{1}{\mathrm{Q_{01}}} + \frac{1}{\mathrm{Q_{02}}} + \frac{\mathrm{R_1}}{\mathrm{Q_1}} + \frac{\mathrm{R_2}}{\mathrm{Q_2}}) + 1$. The inputs to this system are L-ara and C6, which control the M1 switch and M2 switch separately. The theoretical analysis of the Syn-CBS circuit in Fig. 3 and Supplementary Fig. 5 is based on this model. The parameters are, unless otherwise mentioned, $\mathrm{C_{min1}} = 0.003$, $\mathrm{C_{max1}} = 0.1275$, $\mathrm{J_1} = 4.38 * 10^{-4}$, $n = 3$, $\mathrm{v_{01}} = 0.0125$, $\mathrm{v_1} = 0.5$, $\mathrm{d_1} = 0.25$, $\mathrm{C_{min2}} = 0.005$, $\mathrm{C_{max2}} = 0.175$, $\mathrm{J_2} = 4 * 10^{-8}$, $m = 3$, $\mathrm{v_{02}} = 0.0175$, $\mathrm{v_2} = 0.5$, $\mathrm{d_2} = 0.25$, $\mathrm{Q_{01}} = 300$, $\mathrm{Q_1} = 300$, $\mathrm{Q_{02}} = 3.6$, $\mathrm{Q_2} = 3.6$, $\mathrm{Lara} = 0 \sim 10 * 10^{-4}$, and $C6 = 0 \sim 5 * 10^{-8}$. L-ara is set to $9.5 * 10^{-4}$ and C6 is set to $3 * 10^{-8}$ in Fig. 3d.

General mathematical model for the synthetic circuit with resource competition

For a synthetic circuit with multiple genes, we first considered the general model without resource competition. The ordinary differential equations of the mRNA and protein products for each gene follow:

$$\frac{dmRNA_i}{dt} = k_{mi} \cdot R_i - d_{m_i} \cdot mRNA_i$$

$$\frac{dP_i}{dt} = k_{pi} \cdot mRNA_i - d_{Pi} \cdot P_i$$

where R_i is the number of active promoters for each gene that is bound by transcription factors ($DNA_i\colon TF$). For this model, resources such as RNAP and ribosome are not considered yet.

We then considered the transcription resource RNAP in the model. To do so, we needed to consider the binding/unbinding of the RNAP to the active promoter $DNA\colon TF$ in order to start transcription,

$$DNA_i\colon TF + RNAP \xleftrightarrow{k_f^{RNAP}, k_r^{RNAP}} DNA_i\colon TF\colon RNAP \xrightarrow{k'_m} mRNA_i + RNAP\colon TF$$

Here, we assume that the concentrations of the RNA polymerases are constant [2]. It is noted that all the promoters in the synthetic gene circuit compete for the available RNAP within the host cell. Thus, the transcription rate for each gene follows the Michaelis–Menten kinetics with competitive inhibition by all other genes:

$$k'_m \cdot RNAP_t \cdot \frac{R_i/J_{m_i}}{\sum R_i/J_{m_i} \quad + 1} = \frac{k'_{mi} \cdot RNAP_t}{J_{mi}} \cdot \frac{R_i}{\sum R_i/J_{m_i} \quad + 1} = k_{mi} \cdot \frac{R_i}{\sum R_i/J_{m_i} \quad + 1}$$

where $k_{mi} = \frac{k'_{mi} \cdot RNAP_t}{J_{m_i}}$, $J_m = \frac{k'_m + k_r^{RNAP}}{k_f^{RNAP}}$, $RNAP_t$ is the total available RNAP in the host that can be used for the synthetic gene circuits, and J_{m_i} is the Michaelis constant.

Thus, the ODE of mRNA is revised as:

$$\frac{dmRNA_i}{dt} = k_{mi} \cdot \frac{R_i}{\sum R_i/J_{m_i} \quad + 1} - d_{mi} \cdot mRNA_i$$

which can be further simplified to

$$\frac{dmRNA_i}{dt} = \frac{k_{mi} \cdot R_i}{PF_m} - d_{mi} \cdot mRNA_i \,.$$

Here, $PF_m = \sum R_i/J_{m_i} + 1$. Under the condition $R/J_m \ll 1$ (i.e., $RNAP$ is far from saturated), $PF_m = \sum R_i/J_{m_i} + 1$. The ODE of mRNA is the same as the one without RNAP competition (Eq. 1).

We further consider the competition of translation resources such as ribosomes. To do so, we needed to consider the binding/unbinding of the ribosome to each mRNA in order to start translation,

$$mRNA_i + Ribosome \xleftrightarrow{k_f^{Ribosome}, k_r^{Ribosome}} mRNA_i\colon Ribosome \xrightarrow{k'_p} P_i + mRNA_i$$

Here the total concentrations of ribosomes is considered to be constant [2]. All the mRNAs compete for the available ribosome. Thus, the translation rate for each mRNA also follows the Michaelis–Menten kinetics with competitive inhibition by all other mRNAs,

The translation rate of $mRNA_i$ is

$$k'_P Ribosome_t \frac{k_{pi} \cdot mRNA_i}{\sum mRNA_i/J_{p_i} + 1} = k'_P \cdot Ribosome_t \cdot \frac{mRNA/J_p}{mRNA/J_p + 1} = \frac{k_{p_i} \cdot mRNA_i}{\sum mRNA_i/J_{p_i} + 1}$$

where $k_{p_i} = \frac{k'_P \cdot Ribosome_t}{J_{p_i}}$, $J_{p_i} = \frac{k'_P + k_r^{Ribosome}}{k_f^{Ribosome}}$, $Ribosome_t$ is the total available ribosome in the host which can be used for the synthetic gene circuits, and J_{p_i} is the Michaelis constant.

Thus, the ODE of each protein product is:

$$\frac{dP_i}{dt} = k_{pi} \frac{mRNA_i}{\sum mRNA_i/J_{p_i} + 1} - d_{Pi} \cdot P_i$$

which can be further simplified to

$$\frac{dP_i}{dt} = \frac{k_{pi} \cdot mRNA_i}{\mathrm{PF_p}} - d_{Pi} \cdot P_i. \quad (8)$$

Here, $\mathrm{PF_p} = \sum mRNA_i/J_{p_i} + 1$. Under the condition $mRNA/J_p \ll 1$ (i.e., ribosome is far from saturated), $\mathrm{PF_p} = 1$, and the ODE of mRNA is the same as the one without RNAP competition (Eq. 2).

We further simplified equations by elevating the equations of miRNAs

$$\frac{dP}{dt} = \frac{k_{pi} \cdot k_{mi}/d_{mi} \cdot R_i}{PF_m * \mathrm{PF_p}} - d_{Pi} * P_i = \frac{v_{pi} \cdot R_i}{PF_m \cdot \mathrm{PF_p}} - d_{Pi} \cdot P_i \quad (10)$$

Where $v_{pi}(= k_{pi} \cdot \frac{k_{mi}}{d_{m_i}})$ is a lumped parameter that represents the overall gene expression rate. $\mathrm{PF_p} = \sum \frac{k_{mi}/d_{mi} \cdot R_i}{PF_m} \frac{1}{Jp_i} + 1 = \sum \frac{R_i/L_i}{PF_m} + 1$, $L_i = \frac{Jp_i d_{mi}}{k_{mi}} = \frac{d_{mi}\left(k_{r_i}^{ribosome} + k_{p_i}\right)}{k_{mi} k_{f_i}^{ribosome}}$, and $\mathrm{PF_m} = \sum R_i/J_{m_i} + 1$. Thus, $\mathrm{PF_m} \cdot \mathrm{PF_p} = \sum \frac{R_i}{J_{m_i}} + \sum \frac{R_i}{L_i} + 1 = \sum R_i/Q_i + 1$.

Thus, the final simplified general model for the synesthetic gene circuit with resource competition is

$$\frac{dP_i}{dt} = \frac{v_{pi} \cdot R_i}{\mathrm{PF_Q}} - d_{Pi} \cdot P_i \quad (11)$$

where $\mathrm{PF_Q} = \mathrm{PF_m} \cdot \mathrm{PF_p} = \sum R_i/Q_i + 1$, and the new lumped parameter $Q_i = \frac{1}{\frac{1}{J_{mi}} + \frac{1}{L_i}}$ indicates the overall capacity of limited resources in the host cell for synthetic gene circuits. While the ribosome is the main limited resource for synthetic gene circuits [2-5], the contribution of the translational capacity to the lumped parameter Q is more significant than the transcriptional capacity.

Stochastic Models

We also developed stochastic models for all of the synthetic circuits with or without resource competition, which generally can be described as birth-and-death stochastic processes that governed the production and degradation rates in the ODE models. A system size factor Ω is introduced to convert the concentration of each variable X (i.e., x = [x]·Ω). The stochastic transition processes and the corresponding propensity function for all the models are described in Table S4. Gillespie algorithm was used for the stochastic simulation.

Potential landscape computation

For a general two-dimensional system described with the following ordinary differential equations

$$\frac{d[X]}{dt} = f_1([X],[Y]) - g_1([X],[Y]),$$
$$\frac{d[Y]}{dt} = f_2([X],[Y]) - g_2([X],[Y]),$$

where $[X],[Y]$ are the concentration of the two variables, and both $f_i([X],[Y])$ and $g_i([X],[Y])$ represent the production and degradation rates for each variable, respectively.

The corresponding Chemical Master equation (CME)[6] is:

$$\frac{d\,P(X,Y,t)}{dt} = f_1(X-1,Y)P(X-1,Y) + g_1(X+1,Y)P(X+1,Y) + f_2(X,Y-1)P(X,Y-1)$$
$$+ g_2(X,Y+1)P(X,Y+1) - \big(f_1(X,Y) + g_1(X,Y) + f_2(X,Y) + g_2(X,Y)\big)P(X,Y),$$

where X,Y are the number of molecules, and $P(X,Y,t)$ represents the probability of the system in state (X,Y) at time t. The steady-state distribution P_{ss} can be obtained by solving the following equation:

$$0 = f_1(X-1,Y)P_{ss}(X-1,Y) + g_1(X+1,Y)P_{ss}(X+1,Y) + f_2(X,Y-1)P_{ss}(X,Y-1) + g_2(X,Y+1)P_{ss}(X,Y+1) - \big(f_1(X,Y) + g_1(X,Y) + f_2(X,Y) + g_2(X,Y)\big)P_{ss}(X,Y)$$

To numerically solve for the P_{ss}, we rewrote the above equation in matrix form:

$$A \cdot P_{ss} = 0.$$

where A is the transition rate matrix from state $(X+i, Y+j)$ to state (X,Y), defined as

$$A\,(X+i, Y+j \to X,Y) = \begin{cases} -\big(f_1(X,Y) + g_1(X,Y) + f_2(X,Y) + g_2(X,Y)\big) & (i=0, j=0) \\ f_1(X-1,Y) & (i=-1, j=0) \\ g_1(X+1,Y) & (i=\ \ 1, j=0) \\ f_2(X,Y-1) & (i=0, j=-1) \\ g_2(X,Y+1) & (i=0, j=\ \ 1) \\ 0 & otherwise \end{cases}$$

No-flux boundary conditions were used to conserve probability. By solving the above linear equation with the Gauss-Seidel method, we found the steady-state distribution P_{ss} and estimated the potential landscape $U \approx -\ln\,(P_{ss})$ [7].

Reference:

1 Zhang, R. *et al.* Topology-dependent interference of synthetic gene circuit function by growth feedback. *Nat Chem Biol*, doi:10.1038/s41589-020-0509-x (2020).
2 Scott, M., Gunderson, C. W., Mateescu, E. M., Zhang, Z. & Hwa, T. Interdependence of cell growth and gene expression: origins and consequences. *Science* **330**, 1099-1102, doi:10.1126/science.1192588 (2010).
3 Ceroni, F., Algar, R., Stan, G. B. & Ellis, T. Quantifying cellular capacity identifies gene expression designs with reduced burden. *Nat Methods* **12**, 415-418, doi:10.1038/nmeth.3339 (2015).
4 Gyorgy, A. *et al.* Isocost Lines Describe the Cellular Economy of Genetic Circuits. *Biophys J* **109**, 639-646, doi:10.1016/j.bpj.2015.06.034 (2015).
5 Carbonell-Ballestero, M., Garcia-Ramallo, E., Montañez, R., Rodriguez-Caso, C. & Macía, J. Dealing with the genetic load in bacterial synthetic biology circuits: convergences with the Ohm's law. *Nucleic acids research* **44**, 496-507, doi:10.1093/nar/gkv1280 (2016).
6 Hallen, M. *et al.* Computation of Steady-State Probability Distributions in Stochastic Models of Cellular Networks. *PLOS Computational Biology* **7**, e1002209, doi:10.1371/journal.pcbi.1002209 (2011).
7 Wang, J., Xu, L., Wang, E. & Huang, S. The potential landscape of genetic circuits imposes the arrow of time in stem cell differentiation. *Biophysical journal* **99**, 29-39, doi:10.1016/j.bpj.2010.03.058 (2010).
