# Supplementary material for: Winner-takes-all resource competition redirects cascading cell fate transitions
Source: Nat Commun. 2021 Feb 8;12:853. doi: 10.1038/s41467-021-21125-3 (PMC7870843; doi:10.1038/s41467-021-21125-3)
Supplement: Supplementary file 3 — Reporting Summary [file 41467_2021_21125_MOESM3_ESM.pdf]

nature research

Corresponding author(s): Xiaojun Tian

Last updated by author(s): Jan 10, 2021

Reporting Summary

Nature Research wishes to improve the reproducibility of the work that we publish. This form provides structure for consistency and transparency in reporting. For further information on Nature Research policies, see our Editorial Policies and the Editorial Policy Checklist.

Statistics

For all statistical analyses, confirm that the following items are present in the figure legend, table legend, main text, or Methods section.

n/a	Confirmed	
[ ]	[x]	The exact sample size (n) for each experimental group/condition, given as a discrete number and unit of measurement
[ ]	[x]	A statement on whether measurements were taken from distinct samples or whether the same sample was measured repeatedly
[x]	[ ]	The statistical test(s) used AND whether they are one- or two-sided<br>*Only common tests should be described solely by name; describe more complex techniques in the Methods section.*
[x]	[ ]	A description of all covariates tested
[x]	[ ]	A description of any assumptions or corrections, such as tests of normality and adjustment for multiple comparisons
[ ]	[x]	A full description of the statistical parameters including central tendency (e.g. means) or other basic estimates (e.g. regression coefficient) AND variation (e.g. standard deviation) or associated estimates of uncertainty (e.g. confidence intervals)
[x]	[ ]	For null hypothesis testing, the test statistic (e.g. F, t, r) with confidence intervals, effect sizes, degrees of freedom and P value noted<br>*Give P values as exact values whenever suitable.*
[x]	[ ]	For Bayesian analysis, information on the choice of priors and Markov chain Monte Carlo settings
[x]	[ ]	For hierarchical and complex designs, identification of the appropriate level for tests and full reporting of outcomes
[x]	[ ]	Estimates of effect sizes (e.g. Cohen's d, Pearson's r), indicating how they were calculated

Our web collection on statistics for biologists contains articles on many of the points above.

Software and code

Policy information about availability of computer code

Data collection	Flow cytometry data were collected with CFlow 1.0.364.15; Plate reader data were collected with Gen5 2.00.
Data analysis	Flow cytometry data were analyzed with Matlab R2017a (Mathworks); Plate reader data were analyzed with Matlab R2017a (Mathworks).

For manuscripts utilizing custom algorithms or software that are central to the research but not yet described in published literature, software must be made available to editors and reviewers. We strongly encourage code deposition in a community repository (e.g. GitHub). See the Nature Research guidelines for submitting code & software for further information.

Data

Policy information about availability of data

All manuscripts must include a data availability statement. This statement should provide the following information, where applicable:

- Accession codes, unique identifiers, or web links for publicly available datasets
- A list of figures that have associated raw data
- A description of any restrictions on data availability

All relevant data in this study are included in the Source Data files. Any additional data are available from the authors upon reasonable request. Plasmids are available through Addgene (ID 165403-165411).

Field-specific reporting

Life sciences study design

All studies must disclose on these points even when the disclosure is negative.

Sample size	Samples sizes were not calculated statistically, but chosen to be 3 ~ 5 replicates to ensure reproducibility.
Data exclusions	Background non-cellular small particles from the culture media were excluded and doublets discrimination were performed to ensure all the data at the single-cell level.
Replication	All attempts at replication were successful. All experiments were repeated independently at least three times, and mean ± SD were reported.
Randomization	Each sample was started with a colony randomly picked from the corresponding culture plate, and the samples were randomly allocated into experimental groups.
Blinding	No blinding was performed.

Reporting for specific materials, systems and methods

We require information from authors about some types of materials, experimental systems and methods used in many studies. Here, indicate whether each material, system or method listed is relevant to your study. If you are not sure if a list item applies to your research, read the appropriate section before selecting a response.

Materials & experimental systems

n/a	Involved in the study	
☒	☐	Antibodies
☒	☐	Eukaryotic cell lines
☒	☐	Palaeontology and archaeology
☒	☐	Animals and other organisms
☒	☐	Human research participants
☒	☐	Clinical data
☒	☐	Dual use research of concern

Methods

n/a	Involved in the study	
☒	☐	ChIP-seq
☐	☒	Flow cytometry
☒	☐	MRI-based neuroimaging

Flow Cytometry

Plots

Confirm that:

☒ The axis labels state the marker and fluorochrome used (e.g. CD4-FITC).

☒ The axis scales are clearly visible. Include numbers along axes only for bottom left plot of group (a 'group' is an analysis of identical markers).

☒ All plots are contour plots with outliers or pseudocolor plots.

☒ A numerical value for number of cells or percentage (with statistics) is provided.

Methodology

Sample preparation	All samples were diluted 10 folds with Luria-Bertani broth right before loading onto the machine.
Instrument	Accuri C6 flow cytometer from Becton Dickinson
Software	Data were collected with CFlow 1.0.364.15. Percentage data were analyzed with Matlab R2017a (Mathworks).
Cell population abundance	Not applicable
Gating strategy	Cells were gated using FSC-A/FSC-H to eliminate the doublets and non-cellular small particles according to data from the plain LB medium without any cells as a negative control.

☒ Tick this box to confirm that a figure exemplifying the gating strategy is provided in the Supplementary Information.
